# Predictive Value of Plasma Big Endothelin-1 in Adverse Events of Patients With Coronary Artery Restenosis and Diabetes Mellitus: Beyond Traditional and Angiographic Risk Factors

**DOI:** 10.3389/fcvm.2022.854107

**Published:** 2022-05-26

**Authors:** Yue Ma, Tao Tian, Tianjie Wang, Juan Wang, Hao Guan, Jiansong Yuan, Lei Song, Weixian Yang, Shubin Qiao

**Affiliations:** Research Center for Coronary Heart Disease, Fuwai Hospital, National Center for Cardiovascular Diseases, Chinese Academy of Medical Sciences and Peking Union Medical College, Beijing, China

**Keywords:** coronay artery disease, in-stent restenosis, big endothelin-1 (big ET-1), diabetes mellitus, cardiovascular prognosis

## Abstract

**Background:**

Patients with diabetes are a high-risk group for coronary in-stent restenosis (ISR), so it would be valuable to identify biomarkers to predict their prognosis. The plasma big endothelin-1 (big ET-1) level is closely related to cardiovascular adverse events; however, for patients with ISR and diabetes who undergo percutaneous coronary intervention (PCI), whether big ET-1 is independently correlated with prognosis is still uncertain.

**Methods:**

Patients with drug-eluting stent (DES) restenosis who underwent successful re-PCI from January 2017 to December 2018 at the Chinese Academy of Medical Sciences Fuwai Hospital were enrolled and followed up for 3 years. The patients were divided into the tertiles of baseline big ET-1. The primary end points were major adverse cardiovascular events (MACEs): cardiac death, non-fatal myocardial infarction (MI), target lesion revascularization (TLR), and stroke. A Cox multivariate proportional hazard model and the C-statistic were used to evaluate the potential predictive value of big ET-1 beyond traditional and angiographic risk factors.

**Results:**

A total of 1,574 patients with ISR were included in this study, of whom 795 were diabetic. In patients with ISR and diabetes, after an average follow-up of 2.96 ± 0.56 years, with the first tertile of big ET-1 as a reference, the hazard ratio [HR] (95% CI) of MACEs after adjustment for traditional and angiographic risk factors was 1.24 (0.51–3.05) for the second tertile and 2.60 (1.16–5.81) for the third. Big ET-1 improved the predictive value for MACEs over traditional risk factors (C-statistic: 0.64 vs. 0.60, *p* = 0.03). Big ET-1 was not significantly associated with the risk of MACEs in patients without diabetes.

**Conclusion:**

Increased plasma big ET-1 was associated with a higher risk of adverse cardiovascular prognosis independent of traditional and angiographic risk factors, and therefore, it might be used as a predictive biomarker, in patients with ISR and diabetes.

## Introduction

Diabetes mellitus is an important risk factor for the development of cardiovascular diseases, such as coronary artery disease (CAD), cerebrovascular disease, and peripheral artery disease (1, 2). Cardiovascular disease is the main cause of death in diabetic patients (3). The total number of diabetic patients in the world is predicted to increase to 592 million by 2035 (4). Coronary artery in-stent restenosis (ISR), a complication that is unpreventable in patients with percutaneous coronary intervention (PCI), refers to lesions with a vascular diameter stenosis rate ≥50% in the stent and/or within 5 mm of both edges of the stent (5). Although drug-eluting stent (DES) implantation improves the long-term prognosis of patients, ISR is still a serious problem (6, 7). Patients with diabetes are a high-risk group for ISR (8, 9). Therefore, it will be valuable to identify biomarkers with predictive value for the prognosis of patients with ISR and diabetes.

Endothelin-1 (ET-1) is a 21-amino-acid polypeptide that is produced by vascular endothelial cells. It is the most effective vasoconstrictor in the cardiovascular system and has the characteristic of long-lasting action (10). ET-1 and its receptors mediate pathophysiological processes, such as inflammation, oxidative stress, endothelial dysfunction, and insulin resistance, leading to the occurrence and progression of diabetes and atherosclerotic diseases (11). Due to the instability of ET-1 in plasma, its clinical application as a biomarker is limited (12). Big ET-1, as the precursor of ET-1, has a longer half-life and can be used as a surrogate indicator to reflect the ET-1 level (12). Big ET-1 has a useful predictive value in patients with three-vessel CAD, stable CAD, young myocardial infarction (MI), acute myocardial infarction (AMI), and diabetes (13–16); however, little is known about its clinical predictive value in patients with ISR and diabetes, a more vulnerable population of patient with CAD. Therefore, this study is aimed to identify the potential association between big ET-1 and clinical prognosis and determine whether big ET-1 has an incremental effect on risk prediction beyond traditional and angiographic risk factors in patients with ISR and diabetes.

## Methods

### Study Population

**FIGURE 1:**
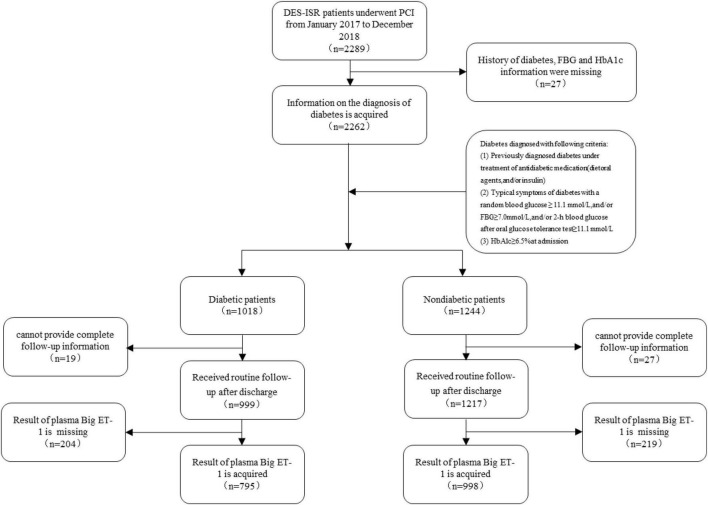
Flow chart of the study population enrolment.

There were 35,649 patients with CAD who underwent successful PCI at the Chinese Academy of Medical Sciences Fuwai Hospital from January 2017 to December 2018, of whom 6.42% of patients (*n* = 2,289) who were diagnosed with DES restenosis were consecutively enrolled in this study. The diagnosis of ISR was based on the presence of lesions in the coronary stent and/or within 5 mm of an edge of the stent, with a vascular diameter stenosis rate ≥50%. According to the angiographic characteristics, it can be divided into four types: type I occurs when the stent or the stent edge is ≤ 10 mm, type II is a diffuse ISR confined to stents >10 mm, type III is a diffuse ISR >10 mm beyond the edge of the stent, and type IV is a completely occlusive ISR (5). Diabetes was diagnosed in patients who met any of the following criteria: fasting blood glucose ≥7.0 mmol/L without any caloric intake for at least 8 h; oral glucose tolerance test (OGTT) with a glucose load of 75 g anhydrous glucose for 2 h; blood glucose ≥11.1 mmol/L; glycosylated hemoglobin ≥6.5%; and random blood glucose ≥11.1 mmol/L in patients with typical symptoms of hyperglycemia (17). The patients were divided into diabetic and non-diabetic groups according to whether they had a diagnosis of diabetes. The exclusion criteria were as follows: a history of diabetes, lack of fasting blood glucose or glycosylated hemoglobin information, lack of plasma big ET-1 test results, and lack of complete follow-up information. The process of selection and exclusion is shown in Figure 1. This study complied with the Declaration of Helsinki and was approved by the ethics review committee. All patients signed an informed consent form.

### Data Collection

In addition to demographic data, patients’ traditional and angiographic risk factors were both collected.

Traditional risk factors include body mass index (BMI), hypertension, hyperlipidemia, diabetes, smoking history, thyroid disease, stroke or transient ischemic attack (TIA), history of peripheral artery disease, chronic kidney failure, and coronary artery bypass grafting (CABG).

Angiographic risk factors include ISR lesion position, ISR angiographic type, pre-Thrombolysis in Myocardial Infarction (TIMI) flow, reference vessel diameter, target lesion length, diameter stenosis rate, and the presence of special types of lesions, such as calcification, occlusion, ostial lesion, thrombus, angulated lesion, and concentric lesion and the number of target lesions. The coronary angiography results were interpreted by two experienced cardiovascular intervention doctors.

The big ET-1 detection method involved drawing 5 ml of fasting venous blood from a vacuum ethylenediaminetetraacetic acid (EDTA) anticoagulant tube, centrifuging at 3,000 r/min for 10 min within 1 h after blood collection, and analyzing the sample by enzyme-linked immunosorbent assay (ELISA) (BIOMEDICA, Austria). The reference value range of big ET-1 was <0.25 pmol/L, and the detection sensitivity was 0.02 pmol/L.

### Follow-Up and End Point Event

The patients were followed up for 3 years by uniformly trained staff through a telephone follow-up or outpatient follow-up. The primary end point was major adverse cardiovascular events (MACEs), which included cardiogenic death, non-fatal MI, target lesion revascularization (TLR), and stroke. Cardiac death was defined as death directly caused by cardiovascular disease. Secondary end points included all-cause death, target vessel revascularization (TVR), stent thrombosis (ST), and hemorrhage. Hemorrhage was defined as intracranial hemorrhage, hemoglobin drop ≥50 g/L, or hematocrit drop ≥15% caused by hemorrhage.

### Statistical Analysis

SPSS 23.0 and R language 3.5.1 statistical software were used to analyze the data. The Kolmogorov-Smirnov normality test was performed on continuous variables. Data with a normal distribution are represented by $\bar{\text{x}}\,\text{±s}$ and were compared between groups using the independent sample *t*-test; data with a non-normal distribution are represented by M (Q1, Q3) and were compared using the Wilcoxon rank-sum test. Categorical variables are expressed as percentages, and the χ^2^ test was used for comparisons between groups. Prior to association analyses, variables with skewed distributions were natural log-transformed. Univariate and multivariate Cox proportional hazard models were used to determine the predictors of the end point event, and the risk is expressed as the hazard ratio (HR) with its 95% confidence interval (CI). All variables with a value of *p* < 0.2 were included in a stepwise Cox regression (*p* < 0.2 as entry criterion and *p* > 0.1 as removal criterion) for identifying potential outcome-specific independent predictors, which were treated as covariates in the ensuing multivariate analyses between big ET-1 and outcomes. The interaction between diabetic status and big ET-1 was tested by adding a product term in the multivariate Cox models. Kaplan-Meier curves were drawn to analyze the survival rate, and the log-rank test was used to compare the difference in survival rate between big ET-1 tertiles. A multivariable-adjusted survival curve was also plotted. A restricted cubic spline was used to analyze the dose-effect relationship between big ET-1 and prognostic events. The C-statistic was calculated to demonstrate the predictive value of big ET-1 for prognostic events when compared with those of traditional and angiographic risk factors. All tests were two-tailed, and differences were considered statistically significant at *p* < 0.05.

## Results

### Baseline Characteristics of the Study Population

The current study finally enrolled 1,793 participants, with a mean age of 60.79 ± 9.77 years and a male proportion of 80.76% (*n* = 1,448). Patients were stratified into two groups according to the diagnosis of diabetes. Patients with diabetes exhibited significantly higher big ET-1 levels than non-diabetic patients (0.23 vs. 0.26, *p* < 0.001). There was no difference between the diabetes group and the non-diabetic group in terms of sex, hyperlipidemia, smoking, thyroid disease, peripheral vascular disease, or other traditional risk factors (*p* > 0.05). The incidence of hypertension, previous stroke, and the levels of N-terminal (NT)-pro-B-type natriuretic peptide (BNP) were higher, and levels of uric acid and high-density lipoprotein cholesterol (HDL-c) were lower in diabetic patients (*p* < 0.05). Both diabetic patients and non-diabetic patients had the left anterior descending (LAD) as the main vessel of the ISR lesion. These groups showed no significant difference in the distribution of angiographic risk factors, such as reference vessel diameter, target lesion length, diameter stenosis rate, special lesion, angiographic type, and pre-TIMI flow (all *p* > 0.05). There was no difference in the medications that were used for the treatment of CAD between the groups (all *p* > 0.05; Table 1). There was no difference in the characteristics between the enrolled and excluded patients (Supplementary Table 1).

**TABLE 1:** Baseline, lesion, and intervention characteristics of patients with coronary artery restenosis.

	Total (*n* = 1,793)	Diabetic patients (*n* = 795)	Non-diabetic patients (*n* = 998)	*P*-value
**Demographic data**				
Age, years	60.79 ± 9.77	61.39 ± 9.03	60.32 ± 10.31	0.0213
Sex, male, n (%)	1,448 (80.76)	631 (79.37)	817 (81.86)	0.1835
BMI, kg/m^2^	26.07 ± 3.14	26.43 ± 3.03	25.79 ± 3.20	<0.0001
**Cardiovascular risk factors, n (%)**				
Hypertension	1,218 (67.97)	594 (74.81)	624 (62.53)	<0.0001
Hyperlipidemia	1,678 (98.36)	750 (98.17)	928 (98.51)	0.5757
Smoking	1,139 (65.54)	493 (63.78)	646 (66.94)	0.1676
**Other disease, n (%)**				
Pre-myocardial infarction	629 (35.08)	287 (36.10)	342 (34.27)	0.4193
Thyroid disease	64 (3.57)	24 (3.02)	40 (4.01)	0.2621
Stroke or TIA	220 (12.27)	116 (14.59)	104 (10.42)	0.0075
Peripheral vascular disease	196 (10.93)	88 (11.07)	108 (10.82)	0.8675
Chronic kidney failure	20 (1.12)	13 (1.64)	7 (0.70)	0.0614
History of CABG	75 (4.18)	39 (4.91)	36 (3.61)	0.1725
**Clinical presenting, n (%)**				0.9738
ACS	833 (46.46)	369 (46.42)	464 (46.49)	
CCS	960 (53.54)	426 (53.58)	534 (53.51)	
**Examination**				
LVEF, %	60.62 ± 7.58	60.21 ± 7.58	60.95 ± 7.58	0.0809
LVDD, mm	49.05 ± 5.42	49.12 ± 5.19	48.98 ± 5.61	0.6448
TnI, ng/L	0.02 (0.00, 0.07)	0.03 (0.00, 0.07)	0.02 (0.00, 0.07)	0.1005
Creatinine, μmol/L	82.52 (72.20, 93.71)	82.65 (72.00, 93.42)	82.27 (73.00, 94.00)	0.6320
Uric acid, μmol/L	350.35 (291.20, 407.85)	335.29 (278.20, 392.00)	359.00 (306.00, 416.35)	<0.0001
TG, mmol/L	1.48 (1.00, 2.05)	1.50 (1.20, 2.10)	1.47 (1.00, 2.04)	0.1566
TC, mmol/L	3.71 (3.20, 4.41)	3.68 (3.20, 4.39)	3.71 (3.20, 4.44)	0.2470
HDL-C, mmol/L	1.06 (1.00, 1.24)	1.03 (0.80, 1.23)	1.08 (1.00, 1.25)	0.0004
LDL-C, mmol/L	2.14 (1.80, 2.71)	2.12 (1.60, 2.71)	2.15 (1.80, 2.71)	0.4423
NT-proBNP, pg/mL	113.20 (51.80, 294.40)	125.10 (54.20, 338.60)	106.80 (49.80, 260.50)	0.0049
Big ET-1, pmol/L	0.24 (0.20, 0.35)	0.26 (0.20, 0.37)	0.23 (0.20, 0.33)	<0.0001
**Big ET-1 in tertiles, n (%)**				
Tertile 1	540 (30.12)	194 (24.40)	346 (34.67)	<0.0001
Tertile 2	615 (34.30)	269 (33.84)	346 (34.67)	<0.0001
Tertile 3	638 (35.58)	332 (41.76)	306 (30.66)	<0.0001
**ISR duration, years**	6.50 (5.40, 7.57)	6.56 (5.60, 7.61)	6.46 (5.20, 7.57)	0.2086
ISR duration type*, n (%)				0.1147
Early ISR	67 (3.74)	36 (4.53)	31 (3.11)	
Later ISR	1,726 (96.26)	759 (95.47)	967 (96.89)	
**ISR lesion position, n (%)**				
LM	44 (2.45)	20 (2.52)	24 (2.40)	0.8801
LAD	733 (40.88)	320 (40.25)	413 (41.38)	0.6284
LCX	247 (13.78)	104 (13.08)	143 (14.33)	0.4466
RCA	571 (31.85)	273 (34.34)	298 (29.86)	0.0431
Graft bypass	8 (0.45)	5 (0.63)	3 (0.30)	0.4785
**ISR lesion features**				
Reference vessel diameter, mm	3.04 ± 0.47	3.02 ± 0.46	3.06 ± 0.47	0.1423
Target lesion length, mm	26.71 ± 19.38	26.55 ± 18.84	26.84 ± 19.82	0.7688
Diameter stenosis rate, %	88.78 ± 9.77	89.02 ± 9.84	88.59 ± 9.71	0.3939
**Special lesion, n (%)**				
Calcification	777 (51.08)	358 (52.34)	419 (50.06)	0.3763
Occlusion	325 (21.05)	141 (20.35)	184 (21.62)	0.5409
Ostial lesion	205 (13.39)	95 (13.85)	110 (13.02)	0.6351
Thrombus	20 (1.34)	10 (1.49)	10 (1.22)	0.6560
Angulated lesion	466 (25.99)	212 (26.67)	254 (25.45)	0.5598
Concentric lesion	271 (15.11)	108 (13.58)	163 (16.33)	0.1066
Diffuse lesion	1,078 (60.12)	461 (57.99)	617 (61.82)	0.1497
**Angiographic type, n (%)**				0.9099
Type I	85 (4.74)	40 (5.03)	45 (4.51)	
Type II	670 (37.37)	300 (37.74)	370 (37.07)	
Type III	708 (39.49)	313 (39.37)	395 (39.58)	
Type IV	330 (18.40)	142 (17.86)	188 (18.84)	
**Pre TIMI flow, n (%)**				0.8481
Class 0	326 (18.18)	140 (17.61)	186 (18.64)	
Class 1	62 (3.46)	26 (3.27)	36 (3.61)	
Class 2	134 (7.47)	57 (7.17)	77 (7.72)	
Class 3	1,271 (70.89)	572 (71.95)	699 (70.04)	
**ISR intervention strategy, n (%)**				
DCB	782 (43.61)	341 (42.89)	441 (44.19)	0.5827
DES	1,011 (56.39)	454 (57.11)	557 (55.81)	0.5827
Non-ISR lesion intervention, n (%)	469 (26.16)	222 (27.92)	247 (24.75)	0.1286
**Number of target lesion, n (%)**				0.0509
1	1,324 (73.84)	573 (72.08)	751 (75.25)	
2	396 (22.09)	180 (22.64)	216 (21.64)	
3	73 (4.07)	42 (5.28)	31 (3.11)	
**Medicine treatment, n (%)**				
Aspirin	1,735 (96.77)	764 (96.10)	971 (97.29)	0.1557
P_2_Y_12_ receptor inhibitor	1,749 (97.55)	774 (97.36)	975 (97.70)	0.6469
Statin	1,737 (96.88)	771 (96.98)	966 (96.79)	0.8206

*Early ISR refers to a duration less than 1 year, and late ISR refers to a duration greater than 1 year; angulated lesion is defined as a lesion with an angle greater than or equal to 45° between the proximal and distal segments; patients were grouped in tertiles according to big ET-1: first (big ET-1 < 0.20 pmol/L), second (0.20 pmol/L ≤ big ET-1 < 0.31 pmol/L), and third (big ET-1 ≥ 0.31 pmol/L).

*LVEF, left ventricular ejection fraction; LVDD, left ventricular diastolic diameter; TnI, troponin I; TG, triglycerides; TC, total cholesterol; HDL-c, high-density lipoprotein cholesterol; LDL-c, low-density lipoprotein cholesterol.*

### Clinical Outcomes

During the average follow-up time of 2.96 ± 0.56 years, 54 patients in the diabetes group (6.79%) experienced MACEs, and 141 patients in the total sample (7.86%) had a secondary end point event. There were 83 patients in the non-diabetic group (8.32%) with MACEs, and 88 patients (8.82%) had secondary end point events. There was no statistically significant difference in the incidence of composite end point events between the two groups (*p* > 0.05), although the incidence of stroke was higher while the incidence of cardiac death was lower in the diabetes group (*p* < 0.05; Table 2).

**TABLE 2:** Follow-up of patients with coronary artery restenosis.

	Total (*n* = 1,793)	Diabetic patients (*n* = 795)	Non-diabetic patients (*n* = 998)	*P*-value
Time of follow-up (years,$\bar{x}$± s)	2.96 ± 0.56	2.96 ± 0.56	2.95 ± 0.55	0.7608
**MACE, n (%)**	137 (7.64)	54 (6.79)	83 (8.32)	0.2275
Cardiac death, n (%)	26 (1.45)	6 (0.75)	20 (2.00)	0.0279
Non-fatal MI, n (%)	12 (0.67)	4 (0.50)	8 (0.80)	0.4413
TLR, n (%)	85 (4.74)	33 (4.15)	52 (5.21)	0.2943
Stroke, n (%)	18 (1.00)	13 (1.64)	5 (0.50)	0.0167
**Secondary endpoints, n (%)**	141 (7.86)	53 (6.67)	88 (8.82)	0.0928
All-cause death, n (%)	42 (2.34)	15 (1.89)	27 (2.71)	0.2549
TVR, n (%)	82 (4.57)	32 (4.03)	50 (5.01)	0.3213
ST*, n (%)	7 (0.39)	1 (0.13)	6 (0.60)	0.1411
Hemorrhage, n (%)	21 (1.17)	8 (1.01)	13 (1.30)	0.5623

*All presented with probable stent thrombosis.

### Kaplan-Meier Analysis

**FIGURE 2:**
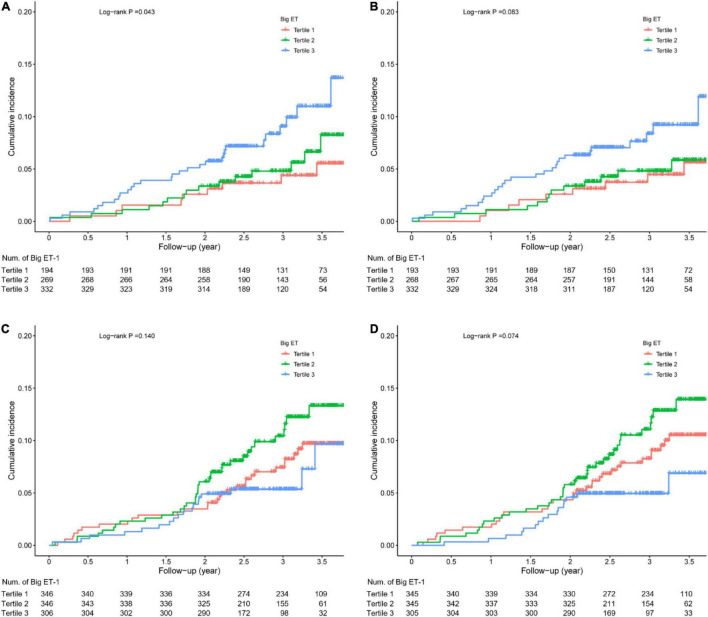
Kaplan-Meier survival analysis. **(A)** Major adverse cardiovascular events (MACE) in diabetic patients; **(B)** secondary end points in diabetic patients; **(C)** MACE in non-diabetic patients; and **(D)** secondary end points in non-diabetic patients.

Among the diabetic patients, the incidence of MACEs in the big ET-1 tertile 3 was higher than that in the other two tertiles (log-rank *p* = 0.043), and the incidence of secondary end point events was slightly but not significantly higher than that in the other two groups (log-rank *p* = 0.083). Among the non-diabetic patients, the incidence of MACEs (log-rank *p* = 0.140) and the incidence of secondary end point events were not significantly different between the big ET-1 tertiles (log-rank *p* = 0.074; Figure 2).

### Stepwise Cox Regression of Traditional and Angiographic Predictors

Univariate Cox regression analysis for traditional and angiographic variables was performed separately for MACEs and secondary end points. Then, all variables with *p* < 0.2 were entered into the multivariable Cox regression analysis for MACEs and secondary end points following the stepwise method for identifying independent predictors. We found that big ET-1 had an independent predictive value in diabetic patients. In addition, angulated lesions, history of CABG, low-density lipoprotein cholesterol (LDL-c), reference vessel diameter, thyroid disease, early ISR, and non-ISR lesion intervention were also associated with the prognosis of ISR patients with diabetes mellitus. All detailed results from the univariate and multivariable analyses are shown in Supplementary Tables 2–5.

### Interaction Between Big ET-1 and Diabetes

When adding big ET-1 (as a continuous variable), a diagnosis of diabetes, and the interaction term of big ET-1 × diagnosis of diabetes (big ET-1 × diabetes) into a Cox regression, after adjusting for traditional and angiographic risk factors, we found that the interaction term was statistically significant for predicting both MACEs and the secondary end points (*p* for interaction < 0.0001). The significant interaction between big ET-1 and the diagnosis of diabetes indicates that diabetes diagnosis could modify the relationship between big ET-1 and the adverse cardiovascular prognosis. When adding big ET-1 as a categorical variable into a Cox regression analysis, after adjusting for traditional and angiographic risk factors, the interaction term was again statistically significant for predicting both MACEs (*p* for interaction = 0.008) and the secondary end points (*p* for interaction = 0.012; Table 3).

**TABLE 3:** Interaction between big endothelin-1 and diabetes in the prognosis of MACEs and secondary end points.

	Wald chi-square value*	*P*-value*	Wald chi-square value**	*P*-value**
**MACE**				
Big ET-1	3.58	0.058	4.56	0.103
Diabetes	8.46	0.004	1.95	0.163
Big ET-1 × Diabetes	13.07	<0.0001	9.61	0.008
Age	3.53	0.06	3.61	0.057
Sex	5.35	0.021	4.87	0.027
BNP	5.28	0.022	6.76	0.009
Diameter stenosis rate	1.83	0.177	1.63	0.201
Thyroid disease	4.53	0.033	4.36	0.037
**Secondary endpoints**				
Big ET-1	2.93	0.087	4.86	0.088
Diabetes	6.23	0.013	1.4	0.236
Big ET-1 × Diabetes	12.55	<0.0001	8.82	0.012
Age	1.07	0.302	1.16	0.282
Sex	3.1	0.078	2.85	0.092
BNP	5.71	0.017	7.48	0.006
Diameter stenosis rate	6.12	0.013	5.78	0.016

*Big endothelin-1 (ET-1) as a continuous variable. **Big ET-1 as a categorical variable, age, sex, and variables found as independent predictors in the overall population were adjusted. Big ET-1 and B-type natriuretic peptide (BNP) were natural log-transformed.

### Relationship Between Big ET-1 and Cardiovascular Prognosis

**FIGURE 3:**
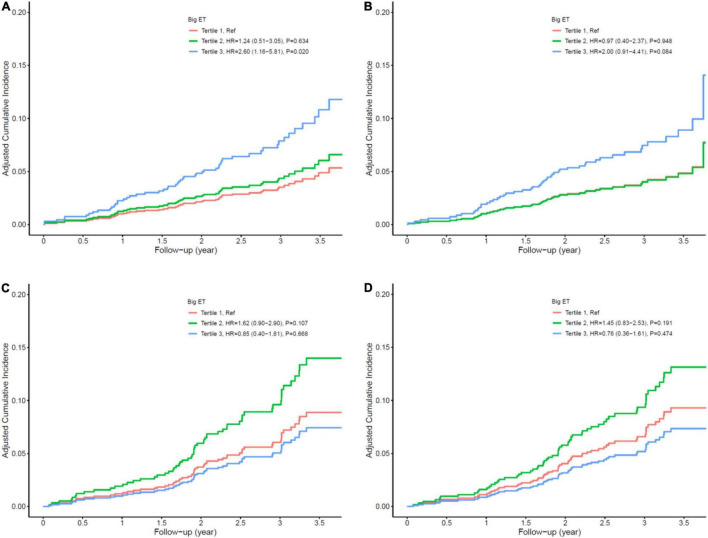
Cox multivariate survival analysis. **(A)** Major adverse cardiovascular events (MACE) in diabetic patients; **(B)** secondary end points in diabetic patients; **(C)** MACE in non-diabetic patients; **(D)** secondary end points in non-diabetic patients.

In the diabetic patients, a one-unit increase in log-transformed big ET-1 was associated with a 105% increase in the risk of MACE (HR = 2.05, 95% CI: 1.36–3.09, *p* = 0.001) and a 98% increase in the risk of secondary end point events (HR = 1.98, 95% CI: 1.29–3.03, *p* = 0.002) after adjusting for age, sex, angulated lesion, history of CABG, LDL-c, reference vessel diameter, thyroid disease, early ISR, and non-ISR lesion intervention as potential traditional and angiographic factors input from the stepwise Cox regression analysis. When we grouped patients into the tertiles of big ET-1 level, compared with the first tertile, the second and third tertiles had HRs of 1.24 (0.51–3.05) and 2.60 (1.16–5.81) for MACE and 0.97 (0.40–2.37) and 2.00 (0.91–4.41) for secondary end point outcomes, respectively, after multivariable adjustment (see Table 4 and Figure 3). The HRs (with their 95% CIs) of all covariates in the model are shown in Supplementary Table 6. The results of the subgroup analysis are shown in Supplementary Figures 1, 2.

**TABLE 4:** Cox proportional hazards models for prognosis in diabetic patients.

	Univariate		Multivariate	
	HR (95% CI)	*P*-value	HR (95% CI)	*P*-value
**MACE**				
Big ET-1*	1.85 (1.26–2.72)	0.002	2.05 (1.36–3.09)	0.001
Big ET-1 tertile 1	Reference	–	Reference	–
Big ET-1 tertile 2	1.35 (0.59–3.09)	0.477	1.24 (0.51–3.05)	0.634
Big ET-1 tertile 3	2.32 (1.09–4.92)	0.029	2.60 (1.16–5.81)	0.02
**Secondary endpoints**				
Big ET-1*	1.77 (1.19–2.63)	0.005	1.98 (1.29–3.03)	0.002
Big ET-1 tertile 1	Reference	–	Reference	–
Big ET-1 tertile 2	1.06 (0.46–2.43)	0.893	0.97 (0.40–2.37)	0.948
Big ET-1 tertile 3	1.93 (0.93–4.04)	0.079	2.00 (0.91–4.41)	0.084

*Big endothelin-1 (ET-1) was natural log-transformed. Major adverse cardiovascular events (MACE) model adjusted for age, sex, angulated lesion, history of coronary artery bypass grafting [CABG], low-density lipoprotein cholesterol [LDL-c], reference vessel diameter, thyroid disease. Secondary end points model adjusted for age, sex, angulated lesion, history of CABG, early ISR, and non-ISR lesion intervention.

Big ET-1 was not significantly associated with the risk of MACEs or secondary end point events in patients without diabetes, whether in univariate or multivariate analysis (all *p* > 0.05; see Table 5 and Figure 3). All results of the multivariate Cox analysis are shown in Supplementary Table 7.

**TABLE 5:** Cox proportional hazards models for prognosis in non-diabetic patients.

	Univariate		Multivariate	
	HR (95% CI)	*P*-value	HR (95% CI)	*P*-value
**MACE**				
Big ET-1*	0.84 (0.55–1.28)	0.415	0.82 (0.50–1.33)	0.418
Big ET-1 tertile 1	Reference	–	Reference	–
Big ET-1 tertile 2	1.42 (0.87–2.32)	0.16	1.62 (0.90–2.90)	0.107
Big ET-1 tertile 3	0.85 (0.47–1.53)	0.581	0.85 (0.40–1.81)	0.668
**Secondary endpoints**				
Big ET-1*	0.74 (0.49–1.13)	0.162	0.82 (0.50–1.33)	0.417
Big ET-1 tertile 1	Reference	–	Reference	–
Big ET-1 tertile 2	1.31 (0.81–2.11)	0.266	1.45 (0.83–2.53)	0.191
Big ET-1 tertile 3	0.67 (0.37–1.23)	0.195	0.76 (0.36–1.61)	0.474

*Big endothelin-1 (ET-1) was natural log-transformed. Major adverse cardiovascular events (MACE) model adjusted for age, sex, left ventricular ejection fraction (LVEF), in-stent restenosis (ISR) duration, hypertension, drug-eluting stent (DES) intervention, total cholesterol (TC), and diameter stenosis rate. Secondary end points model adjusted for age, sex, high-density lipoprotein cholesterol (HDL-c), TC, reference vessel diameter, target lesion length, and diameter stenosis rate.

### Dose-Response Relationship Between big ET-1 and Cardiovascular Prognosis

**FIGURE 4:**
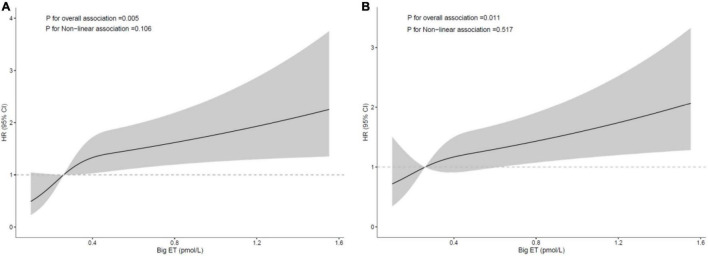
Restricted cubic spline curve for the risk in diabetic patients according to big ET-1; **(A)** major adverse cardiovascular events [MACE; adjusted for age, sex, left ventricular ejection fraction (LVEF), in-stent restenosis (ISR) duration, hypertension, drug-eluting stent (DES) intervention, total cholesterol (TC), and diameter stenosis rate]; **(B)** secondary end points [adjusted for age, sex, high-density lipoprotein cholesterol (HDL-c), TC, reference vessel diameter, target lesion length, and diameter stenosis rate].

The restricted cubic spline analysis after adjustment for traditional and angiographic risk factors showed that the relationships between big ET-1 and MACE and secondary end point events were both linear in the diabetic group. The risk of MACEs and secondary end point events was increased with increasing big ET-1, and the trend was statistically significant (*p* = 0.005 and 0.011, respectively), while the test for a non-linear relationship was not statistically significant (*p* = 0.106 and 0.517, respectively; Figure 4).

### Incremental Predictive Value of Big ET-1 for an Adverse Prognosis

Big ET-1 alone showed a similar predictive value for MACEs as a model incorporating traditional risk factors, with respective C-statistics of 0.60 (0.52–0.68) and 0.60 (0.53–0.68) (*p* for difference = 0.54). Adding big ET-1 to the model with traditional risk factors, a moderate but statistically significant increase in the C-statistic was observed (0.64 (0.56–0.72) vs. 0.60 (0.53–0.68), △C-statistic = 0.03, *p* for difference = 0.03). Similarly, big ET-1 showed additional predictive value for the secondary end point events when added to traditional risk factors [0.67 (0.59–0.75) vs. 0.63 (0.56–0.71), △C-statistic = 0.03, *p* for difference = 0.02]. However, adding big ET-1 to the model with traditional and angiographic risk factors yielded no significant increase in the C-statistic (Table 6).

**TABLE 6:** C-statistics of traditional risk factors and big ET-1 in patients with diabetes.

	MACE			Secondary end points		
	C-statistic (95% CI)	ΔC-statistic	*P*-value	C-statistic (95% CI)	ΔC-statistic	*P*-value
Model 1*	0.60 (0.53–0.68)	Reference	–	0.63 (0.56–0.71)	Reference	–
Model 2*	0.64 (0.56–0.72)	0.033	0.03	0.67 (0.59–0.75)	0.035	0.02
Model 3*	0.68 (0.60–0.75)	Reference	–	0.66 (0.57–0.74)	Reference	–
Model 4*	0.68 (0.60–0.76)	0.005	0.32	0.66 (0.57–0.74)	0.002	0.46

*Model 1: traditional risk factors [age, sex, BMI, smoking, hypertension, hyperlipidemia, stroke or transient ischemic attack (TIA), and left ventricular ejection fraction (LVEF)]; Model 2: traditional risk factors + big endothelin-1 (ET-1); Model 3: traditional and angiographic risk factors [major adverse cardiovascular events (MACE) model adjusted for age, sex, LVEF, in-stent restenosis (ISR) duration, hypertension, DES intervention, total cholesterol (TC), and diameter stenosis rate]. Secondary end points [model adjusted for age, sex, high-density lipoprotein cholesterol (HDL-c), TC, reference vessel diameter, target lesion length, and diameter stenosis rate]; Model 4: traditional and angiographic risk factors + big ET-1.

## Discussion

This study analyzed the predictive value of big ET-1 for the occurrence of adverse cardiovascular events in a cohort of patients with ISR and diabetes mellitus after PCI. The main findings are as follows: (1) patients with ISR and diabetes have higher levels of big ET-1 than non-diabetic patients. (2) Patients with ISR and diabetes who have higher big ET-1 levels have a higher incidence of MACEs than patients with ISR and diabetes with lower levels of big ET-1. (3) Increased plasma big ET-1 level is correlated with a worse prognosis of patients with ISR and diabetes, it has good predictive value even after adjusting for traditional and angiographic risk factors. (4) The level of big ET-1 is linearly correlated with the occurrence of MACEs in patients with ISR and diabetes. (5) The addition of big ET-1 to the traditional cardiovascular risk prediction model significantly improves the ability to stratify prognostic risk for patients with ISR and diabetes.

Although diabetic patients often have other risk factors at the same time, diabetes itself is a powerful independent risk factor for cardiovascular events. Increased blood glucose levels, insulin resistance, hyperlipidemia, inflammation, and thrombosis accelerate the formation of atherosclerosis, leading diabetic patients to become a high-risk group for ISR (1–3, 8, 18).

The level of big ET-1 is closely related to cardiovascular events and is used as a risk predictor of cardiovascular disease (19). The role of big ET-1 in hypertension, diabetes, and myocardial hypertrophy is manifested in poor cardiovascular remodeling, which is caused by an increase in left ventricular mass (20–22). Big ET-1 also helps to predict the risk of congestive heart failure and death in the general population (23, 24). It has predictive value in patients with chronic heart failure (25), and its predictive value is not inferior to those of hemodynamic monitoring indicators (26). In patients with arrhythmia and cardiomyopathy, high big ET-1 level has a certain predictive value for death, malignant arrhythmia, heart transplantation, and other adverse events (27–30).

Big ET-1 has good predictive value for the prognosis of patients with CAD. Zhang et al. observed 6,150 patients with three-vessel CAD and found that a high big ET-1 level is an independent risk factor for long-term mortality, indicating that it has good predictive value in patients with severe CAD (14). Zhou et al. followed up 3,154 patients with stable CAD and 565 patients with AMI who were younger than 35 years old and found that the occurrence of vascular events was closely correlated with the big ET-1 level (13, 15). Yip et al. established a prospective cohort of 186 cases of ST-segment elevation myocardial infarction (STEMI). Big ET-1 was a strong predictor of the independent composite end point of severe deterioration of cardiac function and death within 30 days after emergency PCI (31). Gao et al. in 822 patients with STEMI combined with diabetes, found that the level of big ET-1 had a strong correlation with no reflow after emergency PCI and with the long-term prognosis, indicating that it has a strong predictive value in patients with CAD and diabetes (16). However, there has been no previous research on the relationship between big ET-1 and the prognosis of patients with ISR.

Our study is the first to discover the important predictive value of big ET-1 level for the cardiovascular prognosis, beyond traditional and angiographic risk factors, in patients with ISR and diabetes, though this is in line with the results of previous studies; i.e., high big ET-1 levels predict a poor outcome. The mechanism of action of big ET-1 in the prognosis of patients with ISR and diabetes is still inconclusive, but it may be related to the following factors: (1) overexpression of big ET-1 aggravates diabetes-induced vascular endothelial dysfunction by inducing oxidative stress (32), which clinically can manifest as hyperplasia of the neointima or neovascular atherosclerosis in the stent (33). (2) Big ET-1 can promote the synthesis of inflammatory microglia in diabetic patients (34) and downregulate inflammatory activity to accelerate the progression of atherosclerosis (35). (3) Big ET-1 mediates the increase in nitric oxide production and the uncoupling of calcium signaling to aggravate the contraction of small blood vessels in diabetic patients (36–38), thereby causing angina pectoris due to coronary microcirculation disorder. (4) Big ET-1 alone or together with other agonists can cause platelet activation, and activated platelets can also stimulate endothelial cells to release big ET-1 (39), which leads to the formation of ST. (5) A long-term increase in big ET-1 can cause cerebrovascular accidents in patients with pre-arteriosclerosis (40), and serious cerebrovascular accidents can cause death. These proposed mechanisms are based on reasoning from population characteristics, so the specific pathophysiological mechanisms need to be empirically clarified.

In terms of clinical application value, our findings come from real-world patients with ISR and diabetes. Our study found that traditional and angiographic risk factors, such as an angulated lesion, history of CABG, LDL-C, reference vessel diameter, thyroid disease, early ISR, and non-ISR lesion intervention, had predictive value for adverse cardiovascular events in patients with ISR and diabetes. Moreover, after adjusting for the traditional and angiographic risk factors, big ET-1 still showed independent predictive value, and the increase in big ET-1 was linearly correlated with the increase in the incidence of adverse cardiovascular events. After adding the biomarker big ET-1 to the traditional cardiovascular risk factor model, the C-statistic increased significantly, indicating that big ET-1 can significantly improve the predictive ability of adverse cardiovascular events in diabetic patients. Although the C-statistic was not significantly improved by adding angiographic risk factors, possibly because the sample size was not large enough and the positive rate of angiographic risk factors was low, this does not negate the predictive value of big ET-1, as traditional risk factors are more accessible to clinicians than angiographic risk factors, especially in patients who cannot undergo coronary angiography. The results of our study are of great value in the risk stratification of patients and the detection of high-risk patients (those with big ET-1 > 0.31 pmol/L), so they can guide the formulation of individualized medication choices and revascularization treatment plans for patients, which may improve their life expectancy.

Specific types of DES might yield a more favorable prognosis in terms of target-lesion failure in diabetic patients. The SUGER study showed that Cre8 EVO stents might be superior to Resolute Onyx stents in reducing target lesion failure (41). Drug-coated balloon (DCB) implantation for *de novo* lesions in diabetic patients has demonstrated a lower incidence of TVR than DES implantation (42, 43), which means DCBs are more advantageous in diabetic patients.

This study has some limitations: (1) this was a single-center, observational clinical study, so the external validity of the results is limited. Since the study primarily included Chinese patients, the results and conclusions only apply to Asians and need to be confirmed in other populations in prospective multicenter studies. (2) There was little information on the specific causes of death for the end point events, and the deaths whose causes could not be determined were not all cardiovascular deaths. Prognostic events were adjudicated by physicians but not the clinical events committee (CEC). As the CEC provides a more standardized and independent outcome assessment (44), prognostic events should be adjudicated by the CEC in future study designs. (3) Most studies on big ET-1 and cardiovascular prognosis, such as this study, have been in Chinese patients. Due to the differences in metabolic levels between different races, future studies should be done in multiple centers treating different races. (4) The prognostic analysis of this study was based on the detection of big ET-1 in a single plasma sample. It might be better to take multiple samples and use their average.

## Conclusion

Increased plasma big ET-1 was associated with a higher risk of adverse cardiovascular prognosis independent of traditional and angiographic risk factors, and therefore it might be used as a prognostic/predictive biomarker in patients with ISR and diabetes.

## Data Availability Statement

The raw data supporting the conclusions of this article will be made available by the authors, without undue reservation.

## Ethics Statement

The studies involving human participants were reviewed and approved by the Clinical Research Ethics Committee of Chinese Academy of Medical Sciences Fuwai Hospital. The patients/participants provided their written informed consent to participate in this study. Written informed consent was obtained from the individual(s) for the publication of any potentially identifiable images or data included in this article.

## Author Contributions

YM made substantial contributions to study design, data collection, data analysis, and manuscript writing. JY, LS, WY, and SQ made substantial contributions to study design and intellectual direction. TT, TW, JW, and HG made contributions to data collection and analysis. All authors read and approved the final manuscript.

## Conflict of Interest

The authors declare that the research was conducted in the absence of any commercial or financial relationships that could be construed as a potential conflict of interest.

## Publisher’s Note

All claims expressed in this article are solely those of the authors and do not necessarily represent those of their affiliated organizations, or those of the publisher, the editors and the reviewers. Any product that may be evaluated in this article, or claim that may be made by its manufacturer, is not guaranteed or endorsed by the publisher.

## Abbreviations

ISR: in-stent restenosis
Big ET-1: big endothelin-1
PCI: percutaneous coronary intervention
DES: drug-eluting stent
MI: myocardial infarction
AMI: acute myocardial infarction
MACEs: Major adverse cardiovascular events
TLR: target lesion revascularization
HR: hazard ratio
CI: confidence interval
CAD: coronary artery disease
OGTT: oral glucose tolerance test
HbA1c: glycosylated hemoglobin A1c
BMI: body mass index
TIA: transient ischemic attack
CABG: coronary artery bypass grafting
LVEF: left ventricular ejection fraction
LVDD: left ventricular diastolic diameter
TnI: troponin I
TG: triglycerides
TC: total cholesterol
LDL-c: low-density lipoprotein cholesterol
HDL-c: high-density lipoprotein cholesterol
ELISA: enzyme-linked immunosorbent assay
TVR: target vessel revascularization
ST: stent thrombosis
LM: left main artery
LAD: left anterior descending
LCX: left circumflex
RCA: right coronary artery
CEC: clinical events committee.

## References

[1] Cosentino F Grant PJ Aboyans V Bailey CJ Ceriello A Delgado V 2019 ESC guidelines on diabetes, pre-diabetes, and cardiovascular diseases developed in collaboration with the EASD. *Eur Heart J.* (2020) 41:255–323. 31497854 10.1093/eurheartj/ehz486

[2] Glovaci D Fan W Wong ND. Epidemiology of diabetes mellitus and cardiovascular disease. *Curr Cardiol Rep.* (2019) 21:21. 10.1007/s11886-019-1107-y 30828746

[3] Dal Canto E Ceriello A Ryden L Ferrini M Hansen TB Schnell O Diabetes as a cardiovascular risk factor: an overview of global trends of macro and micro vascular complications. *Eur J Prev Cardiol.* (2019) 26:25–32. 10.1177/2047487319878371 31722562

[4] Guariguata L Whiting DR Hambleton I Beagley J Linnenkamp U Shaw JE. Global estimates of diabetes prevalence for 2013 and projections for 2035. *Diabetes Res Clin Pract.* (2014) 103:137–49. 10.1016/j.diabres.2013.11.002 24630390

[5] Mehran R Dangas G Abizaid AS Mintz GS Lansky AJ Satler LF Angiographic patterns of in-stent restenosis: classification and implications for long-term outcome. *Circulation.* (1999) 100:1872–8. 10.1161/01.cir.100.18.1872 10545431

[6] Bonaa KH Mannsverk J Wiseth R Aaberge L Myreng Y Nygard O Drug-eluting or bare-metal stents for coronary artery disease. *N Engl J Med.* (2016) 375:1242–52. 27572953 10.1056/NEJMoa1607991

[7] Zhu Y Liu K Chen M Liu Y Gao A Hu C Triglyceride-glucose index is associated with in-stent restenosis in patients with acute coronary syndrome after percutaneous coronary intervention with drug-eluting stents. *Cardiovasc Diabetol.* (2021) 20:137. 10.1186/s12933-021-01332-4 34238294 PMC8268452

[8] Scheen AJ Warzee F Legrand VM. Drug-eluting stents: meta-analysis in diabetic patients. *Eur Heart J.* (2004) 25:2167–8. 15571834 10.1016/j.ehj.2004.07.041

[9] Paramasivam G Devasia T Jayaram A UK AR Rao MS Vijayvergiya R In-stent restenosis of drug-eluting stents in patients with diabetes mellitus: clinical presentation, angiographic features, and outcomes. *Anatol J Cardiol.* (2020) 23:28–34. 10.14744/AnatolJCardiol.2019.72916 31911567 PMC7141436

[10] Davenport AP Hyndman KA Dhaun N Southan C Kohan DE Pollock JS Endothelin. *Pharmacol Rev.* (2016) 68:357–418. 26956245 10.1124/pr.115.011833 PMC4815360

[11] Pernow J Shemyakin A Bohm F. New perspectives on endothelin-1 in atherosclerosis and diabetes mellitus. *Life Sci.* (2012) 91:507–16. 10.1016/j.lfs.2012.03.029 22483688

[12] Papassotiriou J Morgenthaler NG Struck J Alonso C Bergmann A. Immunoluminometric assay for measurement of the C-terminal endothelin-1 precursor fragment in human plasma. *Clin Chem.* (2006) 52:1144–51. 10.1373/clinchem.2005.065581 16627560

[13] Zhou BY Gao XY Zhao X Qing P Zhu CG Wu NQ Predictive value of big endothelin-1 on outcomes in patients with myocardial infarction younger than 35 years old. *Per Med.* (2018) 15:25–33. 10.2217/pme-2017-0044 29714117

[14] Zhang C Tian J Jiang L Xu L Liu J Zhao X Prognostic value of plasma big endothelin-1 level among patients with three-vessel disease: a cohort study. *J Atheroscler Thromb.* (2019) 26:959–69. 10.5551/jat.47324 30828008 PMC6845695

[15] Zhou BY Guo YL Wu NQ Zhu CG Gao Y Qing P Plasma big endothelin-1 levels at admission and future cardiovascular outcomes: a cohort study in patients with stable coronary artery disease. *Int J Cardiol.* (2017) 230:76–9. 10.1016/j.ijcard.2016.12.082 28038820

[16] Gao R Wang J Zhang S Yang G Gao Z Chen X. The value of combining plasma D-dimer and endothelin-1 levels to predict no-reflow after percutaneous coronary intervention of ST-segment elevation in acute myocardial infarction patients with a type 2 diabetes mellitus history. *Med Sci Monit.* (2018) 24:3549–56. 10.12659/MSM.908980 29806659 PMC6003259

[17] American Diabetes Association [ADA]. Diagnosis and classification of diabetes mellitus. *Diabetes Care.* (2014) 37:S81–90. 24357215 10.2337/dc14-S081

[18] Flaherty JD Davidson CJ. Diabetes and coronary revascularization. *JAMA.* (2005) 293:1501–8. 15784875 10.1001/jama.293.12.1501

[19] Jankowich M Choudhary G. Endothelin-1 levels and cardiovascular events. *Trends Cardiovasc Med.* (2020) 30:1–8. 30765295 10.1016/j.tcm.2019.01.007

[20] Peng T Li X Hu Z Yang X Ma C. Predictive role of endothelin in left ventricular remodeling of chronic kidney disease. *Ren Fail.* (2018) 40:183–6. 10.1080/0886022X.2018.1455586 29587563 PMC6014341

[21] Valero-Munoz M Li S Wilson RM Boldbaatar B Iglarz M Sam F. Dual Endothelin-A/Endothelin-B receptor blockade and cardiac remodeling in heart failure with preserved ejection fraction. *Circ Heart Fail.* (2016) 9:e003381. 10.1161/CIRCHEARTFAILURE.116.003381 27810862 PMC5584628

[22] Lindman BR Davila-Roman VG Mann DL McNulty S Semigran MJ Lewis GD Cardiovascular phenotype in HFpEF patients with or without diabetes: a RELAX trial ancillary study. *J Am Coll Cardiol.* (2014) 64:541–9. 10.1016/j.jacc.2014.05.030 25104521 PMC4133145

[23] Yokoi K Adachi H Hirai Y Enomoto M Fukami A Ogata K Plasma endothelin-1 level is a predictor of 10-year mortality in a general population: the Tanushimaru study. *Circ J.* (2012) 76:2779–84. 10.1253/circj.cj-12-0469 22971991

[24] Jankowich MD Wu WC Choudhary G. Association of elevated plasma endothelin-1 levels with pulmonary hypertension, mortality, and heart failure in African American individuals: the Jackson heart study. *JAMA Cardiol.* (2016) 1:461–9. 10.1001/jamacardio.2016.0962 27438323

[25] Pousset F Isnard R Lechat P Kalotka H Carayon A Maistre G Prognostic value of plasma endothelin-1 in patients with chronic heart failure. *Eur Heart J.* (1997) 18:254–8. 9043842 10.1093/oxfordjournals.eurheartj.a015228

[26] Pacher R Stanek B Hulsmann M Koller-Strametz J Berger R Schuller M Prognostic impact of big endothelin-1 plasma concentrations compared with invasive hemodynamic evaluation in severe heart failure. *J Am Coll Cardiol.* (1996) 27:633–41. 10.1016/0735-1097(95)00520-x 8606275

[27] Fan P Zhang Y Lu YT Yang KQ Lu PP Zhang QY Prognostic value of plasma big endothelin-1 in left ventricular non-compaction cardiomyopathy. *Heart.* (2021) 107:836–41. 10.1136/heartjnl-2020-317059 33055147 PMC8077223

[28] Wang Y Tang Y Zou Y Wang D Zhu L Tian T Plasma level of big endothelin-1 predicts the prognosis in patients with hypertrophic cardiomyopathy. *Int J Cardiol.* (2017) 243:283–9. 10.1016/j.ijcard.2017.03.162 28587741

[29] Wu S Yang YM Zhu J Ren JM Wang J Zhang H The association between plasma big endothelin-1 levels at admission and long-term outcomes in patients with atrial fibrillation. *Atherosclerosis.* (2018) 272:1–7. 10.1016/j.atherosclerosis.2018.02.034 29529394

[30] Cinar T Hayiroglu M Cicek V Orhan AL. A new marker for ventricular tachyarrhythmias in patients with postinfarction left ventricular aneurysm: big endothelin-1. *Anatol J Cardiol.* (2020) 23:193–4. 10.14744/AnatolJCardiol.2020.46595 32120361 PMC7222630

[31] Yip HK Wu CJ Chang HW Yang CH Yu TH Chen YH Prognostic value of circulating levels of endothelin-1 in patients after acute myocardial infarction undergoing primary coronary angioplasty. *Chest.* (2005) 127:1491–7. 10.1378/chest.127.5.1491 15888819

[32] Idris-Khodja N Ouerd S Mian MOR Gornitsky J Barhoumi T Paradis P Endothelin-1 overexpression exaggerates diabetes-induced endothelial dysfunction by altering oxidative stress. *Am J Hypertens.* (2016) 29:1245–51. 10.1093/ajh/hpw078 27465439 PMC5055737

[33] Nakazawa G Otsuka F Nakano M Vorpahl M Yazdani SK Ladich E The pathology of neoatherosclerosis in human coronary implants bare-metal and drug-eluting stents. *J Am Coll Cardiol.* (2011) 57:1314–22. 10.1016/j.jacc.2011.01.011 21376502 PMC3093310

[34] Abdul Y Jamil S He L Li W Ergul A. Endothelin-1 (ET-1) promotes a proinflammatory microglia phenotype in diabetic conditions. *Can J Physiol Pharmacol.* (2020) 98:596–603. 10.1139/cjpp-2019-0679 32119570 PMC7483816

[35] Wolf D Ley K. Immunity and inflammation in atherosclerosis. *Circ Res.* (2019) 124:315–27. 30653442 10.1161/CIRCRESAHA.118.313591 PMC6342482

[36] Ergul A. Endothelin-1 and diabetic complications: focus on the vasculature. *Pharmacol Res.* (2011) 63:477–82. 10.1016/j.phrs.2011.01.012 21292003 PMC7383935

[37] Abdelhalim MA. Effects of big endothelin-1 in comparison with endothelin-1 on the microvascular blood flow velocity and diameter of rat mesentery in vivo. *Microvasc Res.* (2006) 72:108–12. 10.1016/j.mvr.2006.04.007 17028040

[38] Kalani M. The importance of endothelin-1 for microvascular dysfunction in diabetes. *Vasc Health Risk Manag.* (2008) 4:1061–8. 10.2147/vhrm.s3920 19183753 PMC2605330

[39] Jagroop IA Daskalopoulou SS Mikhailidis DP. Endothelin-1 and human platelets. *Curr Vasc Pharmacol.* (2005) 3:393–9. 16248783 10.2174/157016105774329453

[40] Novo G Sansone A Rizzo M Guarneri FP Pernice C Novo S. High plasma levels of endothelin-1 enhance the predictive value of preclinical atherosclerosis for future cerebrovascular and cardiovascular events: a 20-year prospective study. *J Cardiovasc Med (Hagerstown).* (2014) 15:696–701. 10.2459/JCM.0000000000000121 25000253

[41] Romaguera R Salinas P Gomez-Lara J Brugaletta S Gomez-Menchero A Romero MA Amphilimus- versus zotarolimus-eluting stents in patients with diabetes mellitus and coronary artery disease (SUGAR trial). *Eur Heart J.* (2021). [Epub ahead of print]. 10.1093/eurheartj/ehab790 34735004 PMC8970998

[42] Wohrle J Scheller B Seeger J Farah A Ohlow MA Mangner N Impact of diabetes on outcome with drug-coated balloons versus drug-eluting stents: the BASKET-SMALL 2 trial. *JACC Cardiovasc Interv.* (2021) 14:1789–98. 10.1016/j.jcin.2021.06.025 34412797

[43] Jeger RV Eccleshall S Wan Ahmad WA Ge J Poerner TC Shin ES Drug-coated balloons for coronary artery disease: third report of the international DCB consensus group. *JACC Cardiovasc Interv.* (2020) 13:1391–402. 10.1016/j.jcin.2020.02.043 32473887

[44] Leonardi S Branca M Franzone A McFadden E Piccolo R Juni P Comparison of investigator-reported and clinical event committee-adjudicated outcome events in GLASSY. *Circ Cardiovasc Qual Outcomes.* (2021) 14:e006581. 10.1161/CIRCOUTCOMES.120.006581 33535773

