# Supplementary material for: Predictive Value of Plasma Big Endothelin-1 in Adverse Events of Patients With Coronary Artery Restenosis and Diabetes Mellitus: Beyond Traditional and Angiographic Risk Factors
Source: Front Cardiovasc Med. 2022 May 26;9:854107. doi: 10.3389/fcvm.2022.854107 (PMC9177997; doi:10.3389/fcvm.2022.854107)
Supplement: Supplementary file 1 [file Data_Sheet_1.pdf]

Supplementary information

Table S1 Baseline, lesion and intervention characteristics of enrolled and excluded patients

	Overall (n=2289)	Enrolled Patients (n=1793)	Excluded Patients (n=496)	P value
Demographic data				
Age, years	60.75±9.81	60.79±9.77	60.61±9.95	0.7059
Sex, male, n(%)	1837(80.25)	1448(80.76)	389(78.43)	0.2484
BMI, kg/m2	26.04±3.20	26.07±3.14	25.95±3.39	0.4654
Cardiovascular risk factors, n(%)				
Hypertension	1553(67.99)	1218(67.97)	335(68.09)	0.9595
Dyslipidemia	2143(98.53)	1678(98.36)	465(99.15)	0.2091
Smoking	1445(65.00)	1139(65.54)	306(63.09)	0.3187
Other disease, n(%)				
Stroke	286(12.49)	220(12.27)	66(13.31)	0.5367
History of CABG	98(4.28)	75(4.18)	23(4.64)	0.6583
Thyroid disease	76(3.32)	64(3.57)	12(2.42)	0.2058
Peripheral vascular disease	242(10.57)	196(10.93)	46(9.27)	0.2881
Examination				
Big ET-1	0.24(0.20,0.35)	0.24(0.20,0.35)	0.28(0.20,0.38)	0.2220
TC	3.72(3.20,4.44)	3.71(3.20,4.41)	3.81(3.20,4.54)	0.1858
LDL-C	2.15(1.80,2.73)	2.14(1.80,2.71)	2.20(1.80,2.81)	0.2968
HDL-C	1.06(1.00,1.24)	1.06(1.00,1.24)	1.04(0.80,1.24)	0.5922
TG	1.49(1.00,2.06)	1.48(1.00,2.05)	1.51(1.20,2.06)	0.5319
Creatinine	82.22(72.40,93.15)	82.52(72.20,93.71)	81.34(72.80,91.17)	0.2328
Uric acid	349.53(291.40,408.22)	350.35(291.20,407.85)	343.52(293.80,410.32)	0.7348
BNP	112.20(49.80,303.00)	113.20(51.80,294.40)	104.90(41.80,333.65)	0.4270

Table S2. Unadjusted HR of covariates in all subjects

	Reference	β for MACE	Standard Error for MACE	P for MACE	Included for stepwise regression	β for SACE	Standard Error for SACE	P for SACE	Included for stepwise regression
Age		0.002	0.009	0.808		0.008	0.009	0.371	
Sex	Female	-0.3	0.2	0.133	Yes	-0.171	0.207	0.408	
Smoking	No	-0.002	0.181	0.99		0.279	0.191	0.145	Yes
BMI		0.018	0.029	0.542		-0.003	0.03	0.909	
Hypertension	No	0.212	0.189	0.263		-0.053	0.181	0.769	
Big ET-1*		0.181	0.149	0.226		0.065	0.154	0.673	
TC*		-0.113	0.387	0.77		-0.402	0.383	0.294	
LDL-c*		-0.051	0.257	0.844		-0.311	0.256	0.224	

	Reference	β for MACE	Standard Error for MACE	P for MACE	Included for stepwise regression	β for SACE	Standard Error for SACE	P for SACE	Included for stepwise regression
HDL-c*		-0.128	0.381	0.737		0.416	0.374	0.266	
TG*		0.138	0.183	0.45		-0.06	0.184	0.746	
Creatinine*		0.607	0.37	0.101	Yes	0.707	0.359	0.049	Yes
Uric acid*		-0.349	0.334	0.297		-0.194	0.336	0.564	
TnI*		0.067	0.053	0.202		0.061	0.054	0.256	
BNP*		0.172	0.069	0.013	Yes	0.17	0.07	0.015	Yes
LVDD		0.024	0.017	0.172	Yes	0.024	0.017	0.177	Yes
LVEF		-0.024	0.011	0.036	Yes	-0.014	0.012	0.244	
History of Stroke	No	0.139	0.247	0.575		0.265	0.237	0.264	
History of CABG	No	0.52	0.345	0.131	Yes	0.533	0.345	0.123	Yes
Reference vessel diameter		-0.026	0.198	0.894		-0.103	0.2	0.607	
Target lesion length		0.001	0.005	0.864		0.005	0.004	0.251	
Diameter stenosis rate		0.018	0.01	0.076	Yes	0.03	0.011	0.007	Yes
Calcification	No	0.216	0.182	0.235		0.186	0.185	0.316	
Occlusion	No	-0.059	0.224	0.793		0.135	0.216	0.531	
Ostial lesion	No	0.114	0.255	0.656		0.089	0.262	0.734	
Thrombus	No	-0.386	1.003	0.7		-0.374	1.004	0.71	
Thyroid disease	No	0.612	0.365	0.093	Yes	0.455	0.388	0.241	
Peripheral vascular disease	No	0.32	0.242	0.187	Yes	0.273	0.248	0.27	
CKF	No	-0.4	1.003	0.69		-0.398	1.003	0.691	
Asprin	No	-0.273	0.418	0.514		-0.462	0.388	0.234	
P_2Y_{12}	No	-0.221	0.507	0.663		-0.219	0.508	0.667	
Statin	No	-0.345	0.418	0.409		-0.347	0.418	0.407	
RCA	No	-0.013	0.184	0.942		-0.073	0.187	0.696	
LAD	No	0.101	0.172	0.559		0.088	0.173	0.612	
LCX	No	0.203	0.233	0.384		0.268	0.229	0.242	
LM	No	0.466	0.456	0.306		0.223	0.508	0.66	
Graft bypass	No	-12.008	510.005	0.981		-12.008	513.858	0.981	
DES intervention	No	-0.135	0.172	0.434		-0.08	0.173	0.646	

	Reference	β for MACE	Standard Error for MACE	P for MACE	Included for stepwise regression	β for SACE	Standard Error for SACE	P for SACE	Included for stepwise regression
Non-ISR lesion intervention	No	0.08	0.193	0.677		0.05	0.195	0.796	
ACS	No	0.047	0.172	0.786		0.156	0.172	0.365	
Pre MI	No	0.138	0.176	0.434		-0.12	0.184	0.514	
ISR duration		-0.028	0.037	0.44		-0.022	0.036	0.542	
Early ISR	No	0.659	0.365	0.071	Yes	0.501	0.388	0.197	Yes
ISR Type2	Type 1	-0.117	0.404	0.772		-0.287	0.408	0.482	
ISR Type3	Type 1	-0.101	0.402	0.802		-0.017	0.4	0.967	
ISR Type4	Type 1	-0.113	0.428	0.791		0.014	0.423	0.974	
Pre TIMI 1	TIMI 0	0.292	0.423	0.489		0.359	0.398	0.367	
Pre TIMI 2	TIMI 0	-0.002	0.348	0.995		-0.282	0.369	0.445	
Pre TIMI 3	TIMI 0	-0.186	0.217	0.391		-0.281	0.212	0.184	Yes
Defuse lesion	Limitation	0.265	0.323	0.413		0.574	0.353	0.104	Yes
Concentric lesion	Eccentricity	-0.007	0.242	0.976		-0.131	0.253	0.605	
Angulated lesion	No	0.34	0.183	0.064	Yes	0.425	0.182	0.019	Yes
Number of lesion 2	1	0.013	0.211	0.951		0.012	0.211	0.954	
Number of lesion 3	1	0.371	0.368	0.313		0.227	0.391	0.562	

*These variables were natural log-transformed

Table S3. Unadjusted HR of covariates in diabetic patients

	Reference	β for MACE	Standard Error for MACE	P for MACE	Included for stepwise regression	β for SACE	Standard Error for SACE	P for SACE	Included for stepwise regression
Age		0.007	0.015	0.651		0.015	0.015	0.348	
Sex	Female	-0.117	0.328	0.722		-0.077	0.341	0.821	
Smoking	No	-0.256	0.279	0.359		-0.02	0.295	0.946	
BMI		0.027	0.048	0.57		0.019	0.05	0.7	
Hypertension	No	-0.204	0.299	0.494		-0.379	0.298	0.204	
Big ET-1*		0.616	0.195	0.002	Yes	0.569	0.202	0.005	Yes
TC*		1.172	0.579	0.043	Yes	-0.071	0.633	0.911	
LDL-c*		0.904	0.394	0.022	Yes	0.028	0.42	0.947	

	Reference	β for MACE	Standard Error for MACE	P for MACE	Included for stepwise regression	β for SACE	Standard Error for SACE	P for SACE	Included for stepwise regression
HDL-c*		-0.601	0.621	0.333		-0.212	0.622	0.733	
TG*		0.299	0.281	0.288		0.054	0.296	0.856	
Creatinine*		0.748	0.478	0.117	Yes	1.085	0.398	0.006	Yes
Uric acid*		-0.393	0.511	0.442		-0.059	0.543	0.913	
TnI*		0.111	0.073	0.13	Yes	0.079	0.078	0.307	
BNP*		0.28	0.104	0.007	Yes	0.319	0.109	0.003	Yes
LVDD		-0.017	0.031	0.583		-0.007	0.032	0.827	
LVEF		-0.018	0.018	0.323		-0.011	0.02	0.573	
History of Stroke	No	0.048	0.384	0.901		0.092	0.386	0.812	
History of CABG	No	1.002	0.434	0.021	Yes	1.067	0.435	0.014	Yes
Reference vessel diameter		0.563	0.299	0.06	Yes	0.542	0.307	0.078	Yes
Target lesion length		-0.001	0.008	0.905		0.001	0.008	0.929	
Diameter stenosis rate		0.012	0.016	0.441		0.03	0.018	0.1	Yes
Calcification	No	0.215	0.289	0.457		-0.002	0.302	0.994	
Occlusion	No	-0.202	0.368	0.583		0.082	0.359	0.818	
Ostial lesion	No	-0.119	0.436	0.785		0.202	0.412	0.624	
Thrombus	No	0.356	1.01	0.725		0.448	1.012	0.658	
Thyroid disease	No	1.045	0.52	0.045	Yes	0.299	0.722	0.679	
Peripheral vascular disease	No	0.48	0.366	0.19	Yes	0.569	0.368	0.122	Yes
CKF	No	0.232	1.01	0.819		0.276	1.011	0.785	
Asprin	No	0.173	0.722	0.811		0.106	0.723	0.883	
P_2Y_{12}	No	0.351	1.009	0.728		0.3	1.01	0.767	
Statin	No	-0.181	0.721	0.802		-0.218	0.722	0.763	
RCA	No	0.12	0.282	0.669		0.114	0.29	0.695	
LAD	No	0.07	0.275	0.799		-0.234	0.293	0.425	
LCX	No	0.44	0.351	0.21		0.648	0.341	0.058	Yes
LM	No	0.458	0.721	0.525		0.501	0.722	0.488	
Graft bypass	No	-12.01	716.627	0.987		-12.01	732.636	0.987	
DES intervention	No	0.163	0.284	0.566		0.07	0.289	0.809	

	Reference	β for MACE	Standard Error for MACE	P for MACE	Included for stepwise regression	β for SACE	Standard Error for SACE	P for SACE	Included for stepwise regression
Non-ISR lesion intervention	No	0.468	0.283	0.099	Yes	0.574	0.289	0.047	Yes
ACS	No	-0.006	0.276	0.983		0.101	0.283	0.721	
Pre MI	No	0.475	0.275	0.085	Yes	-0.01	0.295	0.974	
ISR duration		0.022	0.055	0.697		0.029	0.056	0.61	
Early ISR	No	0.986	0.471	0.036	Yes	0.742	0.522	0.155	Yes
ISR Type2	Type 1	0.362	0.741	0.626		-0.326	0.63	0.605	
ISR Type3	Type 1	0.382	0.738	0.605		-0.111	0.618	0.857	
ISR Type4	Type 1	0.244	0.781	0.755		-0.084	0.659	0.898	
Pre TIMI 1	TIMI 0	0.854	0.592	0.149	Yes	0.842	0.592	0.155	Yes
Pre TIMI 2	TIMI 0	0.143	0.56	0.799		-0.469	0.675	0.487	
Pre TIMI 3	TIMI 0	-0.109	0.359	0.762		-0.154	0.361	0.67	
Defuse lesion	Limitation	1.102	0.739	0.135	Yes	1.825	1.026	0.075	Yes
Concentric lesion	Eccentricity	-0.19	0.434	0.662		-0.337	0.471	0.474	
Angulated lesion	No	0.597	0.28	0.033	Yes	0.778	0.284	0.006	Yes
Number of lesion 2	1	0.394	0.311	0.205		0.565	0.31	0.068	Yes
Number of lesion 3	1	0.725	0.48	0.131	Yes	0.611	0.533	0.252	

*These variables were natural log-transformed

Table S4. Unadjusted HR of covariates in nondiabetic patients

	Reference	β for MACE	Standard Error for MACE	P for MACE	Included for stepwise regression	β for SACE	Standard Error for SACE	P for SACE	Included for stepwise regression
Age		0.001	0.011	0.944		0.006	0.011	0.567	
Sex	Female	-0.442	0.253	0.08	Yes	-0.261	0.26	0.315	
Smoking	No	0.158	0.242	0.515		0.459	0.256	0.073	Yes
BMI		0.018	0.036	0.631		-0.008	0.037	0.829	
Hypertension	No	0.514	0.245	0.036	Yes	0.199	0.229	0.384	
Big ET-1*		-0.173	0.212	0.415		-0.296	0.212	0.162	Yes
TC*		-1.018	0.498	0.041	Yes	-0.614	0.477	0.198	Yes
LDL-c*		-0.69	0.322	0.032	Yes	-0.517	0.32	0.106	Yes

	Reference	β for MACE	Standard Error for MACE	P for MACE	Included for stepwise regression	β for SACE	Standard Error for SACE	P for SACE	Included for stepwise regression
HDL-c*		0.059	0.489	0.904		0.669	0.475	0.159	Yes
TG*		0.049	0.24	0.839		-0.103	0.235	0.661	
Creatinine*		0.443	0.569	0.436		0.155	0.558	0.782	
Uric acid*		-0.435	0.45	0.334		-0.455	0.435	0.297	
TnI*		0.021	0.076	0.779		0.042	0.074	0.568	
BNP*		0.101	0.092	0.275		0.087	0.09	0.332	
LVDD		0.045	0.021	0.029	Yes	0.038	0.021	0.066	Yes
LVEF		-0.029	0.014	0.047	Yes	-0.017	0.015	0.253	
History of Stroke	No	0.241	0.324	0.457		0.43	0.301	0.154	Yes
History of CABG	No	-0.019	0.588	0.974		-0.037	0.588	0.949	
Reference vessel diameter		-0.467	0.262	0.075	Yes	-0.579	0.262	0.027	Yes
Target lesion length		0.002	0.006	0.769		0.007	0.005	0.181	Yes
Diameter stenosis rate		0.023	0.013	0.09	Yes	0.031	0.014	0.026	Yes
Calcification	No	0.226	0.235	0.337		0.313	0.235	0.182	Yes
Occlusion	No	0.026	0.283	0.928		0.172	0.271	0.526	
Ostial lesion	No	0.26	0.316	0.41		0.02	0.34	0.954	
Thrombus	No	-13.019	760.011	0.986		-13.019	753.65	0.986	
Thyroid disease	No	0.275	0.513	0.592		0.495	0.461	0.283	
Peripheral vascular disease	No	0.213	0.324	0.512		0.079	0.337	0.814	
CKF	No	-12.012	513.458	0.981		-12.012	508.229	0.981	
Asprin	No	-0.644	0.513	0.209		-0.889	0.461	0.054	Yes
P_2Y_{12}	No	-0.53	0.588	0.367		-0.495	0.588	0.399	
Statin	No	-0.436	0.513	0.395		-0.413	0.512	0.42	
RCA	No	-0.096	0.245	0.696		-0.184	0.248	0.458	
LAD	No	0.117	0.221	0.595		0.27	0.217	0.214	
LCX	No	0.035	0.312	0.91		0.006	0.312	0.985	
LM	No	0.476	0.588	0.418		0.004	0.716	0.995	
Graft bypass	No	-11.006	462.085	0.981		-11.006	461.29	0.981	
DES intervention	No	-0.319	0.22	0.147	Yes	-0.166	0.218	0.445	

	Reference	β for MACE	Standard Error for MACE	P for MACE	Included for stepwise regression	β for SACE	Standard Error for SACE	P for SACE	Included for stepwise regression
Non-ISR lesion intervention	No	-0.209	0.272	0.443		-0.32	0.278	0.249	
ACS	No	0.078	0.22	0.723		0.186	0.217	0.39	
Pre MI	No	-0.088	0.234	0.709		-0.176	0.235	0.455	
ISR duration		-0.064	0.048	0.19	Yes	-0.054	0.048	0.258	
Early ISR	No	0.326	0.588	0.579		0.298	0.588	0.612	
ISR Type2	Type 1	-0.421	0.483	0.384		-0.318	0.537	0.554	
ISR Type3	Type 1	-0.408	0.481	0.396		-0.011	0.526	0.983	
ISR Type4	Type 1	-0.345	0.512	0.501		0.028	0.553	0.96	
Pre TIMI 1	TIMI 0	-0.176	0.624	0.778		0.008	0.548	0.988	
Pre TIMI 2	TIMI 0	-0.105	0.445	0.814		-0.22	0.439	0.617	
Pre TIMI 3	TIMI 0	-0.226	0.272	0.405		-0.358	0.262	0.171	Yes
Defuse lesion	Limitation	-0.066	0.362	0.855		0.211	0.377	0.575	
Concentric lesion	Eccentricity	0.068	0.293	0.817		-0.058	0.301	0.848	
Angulated lesion	No	0.165	0.245	0.501		0.192	0.241	0.426	
Number of lesion 2	1	-0.261	0.294	0.375		-0.384	0.302	0.204	
Number of lesion 3	1	0.082	0.59	0.889		0.02	0.59	0.973	

*These variables were natural log-transformed

Table S5. Results of stepwise Cox regression for MACE and SACE

Variables (Overall)	β (Overall)	Standard Error (Overall)	P (Overall)	Variables (DM)	β (DM)	Standard Error (DM)	P (DM)	Variables (Non-DM)	β (Non-DM)	Standard Error (Non-DM)	P (Non-DM)
MACE											
BNP*	0.275	0.093	0.003	Angulated lesion	0.927	0.411	0.024	LVEF	-0.03	0.018	0.09
Diameter stenosis rate	0.032	0.016	0.042	History of CABG	1.635	0.53	0.002	ISR duration	-0.185	0.065	0.005
Thyroid disease	1.402	0.404	0.001	Big ET-1*	0.812	0.293	0.006	Hypertension	0.683	0.353	0.053

Variables (Overall)	β (Overall)	Standard Error (Overall)	P (Overall)	Variables (DM)	β (DM)	Standard Error (DM)	P (DM)	Variables (Non-DM)	β (Non-DM)	Standard Error (Non-DM)	P (Non-DM)
				LDL-c*	1.629	0.559	0.004	DES interventio n	-0.594	0.316	0.06
				Reference vessel diameter	1.211	0.449	0.007	TC*	-1.56	0.637	0.014
				Thyroid disease	1.661	0.638	0.009	Diameter stenosis rate	0.045	0.019	0.016
SACE											
BNP*	0.256	0.092	0.005	Angulated lesion	0.655	0.34	0.054	HDL-c*	1.518	0.642	0.018
Diameter stenosis rate	0.053	0.017	0.002	History of CABG	1.052	0.489	0.032	TC*	-1.318	0.666	0.048
	.	.	.	Early ISR	1.096	0.615	0.075	Reference vessel diameter	-0.748	0.358	0.037
	.	.	.	Big ET-1*	1.048	0.243	0	Target lesion length	0.016	0.007	0.022
	.	.	.	Non-ISR lesion interventio n	0.811	0.349	0.02	Diameter stenosis rate	0.04	0.02	0.041

*These variables were natural log-transformed

Table S6 Cox multivariate proportional hazards models for the prognosis of diabetic patients

	HR(95% CI)	P value
MACE		
Big ET-1 Tertile1	Reference	-
Big ET -1 Tertile2	1.24 (0.51-3.05)	0.634
Big ET -1 Tertile3	2.60 (1.16-5.81)	0.02
Age	1.00 (0.97-1.03)	0.881
Sex	1.04 (0.52-2.09)	0.91
Angulated lesion	1.45 (0.81-2.58)	0.211
History of CABG	2.45 (1.02-5.91)	0.046
LDL-c*	2.63 (1.14-6.10)	0.024
Reference vessel diameter	1.86 (1.02-3.40)	0.043
Thyroid disease	2.78 (0.99-7.81)	0.053
Secondary end points		
Big ET-1 Tertile1	Reference	-

	HR(95% CI)	P value
Big ET -1 Tertile2	0.97 (0.40-2.37)	0.948
Big ET -1 Tertile3	2.00 (0.91-4.41)	0.084
Age	1.01 (0.98-1.05)	0.472
Sex	1.03 (0.50-2.13)	0.944
Angulated lesion	2.04 (1.12-3.71)	0.019
History of CABG	3.03 (1.25-7.34)	0.014
Early ISR	3.02 (1.06-8.57)	0.038
Non-ISR lesion intervention	2.01 (1.10-3.69)	0.024

*These variables were natural log-transformed

Table S7 Cox multivariate proportional hazards models for the prognosis of nondiabetic patients

	HR(95% CI)	P value
MACE		
Big ET-1 Tertile1	Reference	-
Big ET -1 Tertile2	1.62 (0.90-2.90)	0.107
Big ET -1 Tertile3	0.85 (0.40-1.81)	0.668
Age	1.00 (0.97-1.03)	0.839
Sex	0.70 (0.36-1.33)	0.273
LVEF	0.97 (0.93-1.00)	0.072
ISR duration	0.91 (0.80-1.03)	0.143
Hypertension	1.31 (0.75-2.27)	0.338
DES intervention	0.53 (0.31-0.88)	0.015
TC*	0.37 (0.13-1.08)	0.069
Diameter stenosis rate	1.03 (1.00-1.06)	0.05
Secondary end points		
Big ET-1 Tertile1	Reference	-
Big ET -1 Tertile2	1.45 (0.83-2.53)	0.191
Big ET -1 Tertile3	0.76 (0.36-1.61)	0.474
Age	1.00 (0.97-1.02)	0.715
Sex	0.84 (0.45-1.55)	0.569
HDL-c*	2.88 (0.91-9.13)	0.072
TC*	0.46 (0.16-1.36)	0.161
Reference vessel diameter	0.54 (0.30-0.97)	0.038
Target lesion length	1.01 (1.00-1.02)	0.07
Diameter stenosis rate	1.03 (1.00-1.06)	0.096

*These variables were natural log-transformed

	Cases/ Participants	HR (95% CI)		*P* value	*P* value for interaction
**Sex**					
Women	12/164		1.77 (0.54-5.83)	0.347	0.366
Men	42/631		2.00 (1.27-3.13)	0.003	
**Age**					
<60 years	21/331		1.86 (0.94-3.71)	0.077	0.573
>=60 years	33/464		2.00 (1.21-3.31)	0.007	
**Current/former smoker**					
No	23/280		1.26 (0.63-2.48)	0.513	0.183
Yes	31/515		2.43 (1.50-3.94)	0.000	
**Overweight or obesity**					
No	6/135		1.45 (0.33-6.28)	0.620	0.535
Yes	48/660		2.14 (1.39-3.29)	0.001	
**Hypertension**					
No	16/200		1.90 (0.88-4.09)	0.102	0.595
Yes	38/595		2.41 (1.42-4.11)	0.001	
**Hyperlipidemia**					
No	3/14		NA	NA	NA
Yes	51/781		1.98 (1.30-3.04)	0.002	
**Stroke**					
No	46/679		2.36 (1.54-3.62)	0.000	0.111
Yes	8/116		0.76 (0.20-2.92)	0.689	
**Peripheral vascular diseases**					
No	45/707		2.18 (1.42-3.34)	0.000	NA
Yes	9/88		NA	NA	
**Using p2y12 inhibitors**					
No	1/21		NA	NA	NA
Yes	53/774		2.06 (1.37-3.12)	0.001	
**Using statin**					
No	2/24		NA	NA	NA
Yes	52/771		2.11 (1.40-3.18)	0.000	
**Clinical presentation on admission**					
CCS	30/426		2.04 (1.18-3.52)	0.01	0.851
ACS	24/369		2.26 (1.18-4.34)	0.014	
**Previous myocardial infraction**					
No	29/508		1.91 (1.10-3.32)	0.021	0.424
Yes	25/287		2.75 (1.41-5.36)	0.003	

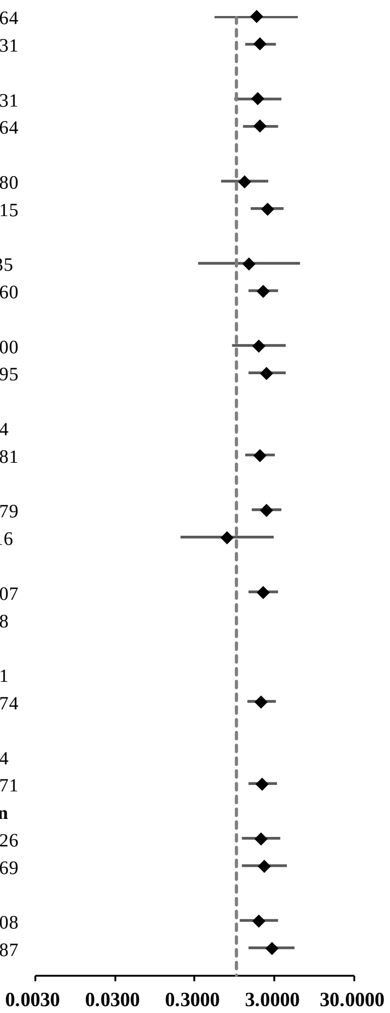

Fig. S1 Cox proportional hazards analysis between big ET-1 and MACEs in various stratifications.

	Cases/ Participants	HR (95% CI)	*P* value	*P* value for interaction
**Sex**				
Women	11/164	2.44 (0.71-8.45)	0.158	0.714
Men	42/631	2.01 (1.26-3.19)	0.003	
**Age**				
<60 years	21/331	1.85 (0.91-3.77)	0.090	0.659
>=60 years	32/464	2.10 (1.20-3.66)	0.009	
**Current/former smoker**				
No	19/280	0.98 (0.43-2.25)	0.959	0.066
Yes	34/515	2.28 (1.42-3.65)	0.001	
**Overweight or obesity**				
No	5/135	NA	NA	NA
Yes	48/660	1.94 (1.26-3.00)	0.003	
**Hypertension**				
No	17/200	2.03 (0.90-4.55)	0.088	0.567
Yes	36/595	2.38 (1.38-4.10)	0.002	
**Hyperlipidemia**				
No	4/14	NA	NA	NA
Yes	49/781	2.00 (1.27-3.17)	0.003	
**Stroke**				
No	45/679	2.35 (1.48-3.74)	0.000	NA
Yes	8/116	NA	NA	
**Peripheral vascular diseases**				
No	44/707	1.91 (1.20-3.03)	0.006	NA
Yes	9/88	NA	NA	
**Using p2y12 inhibitors**				
No	1/21	NA	NA	NA
Yes	52/774	1.99 (1.29-3.08)	0.002	
**Using statin**				
No	2/24	NA	NA	NA
Yes	51/771	1.93 (1.23-3.01)	0.004	
**Clinical presentation on admission**				
CCS	28/426	2.34 (1.36-4.01)	0.002	0.414
ACS	25/369	1.56 (0.75-3.26)	0.237	
**Previous myocardial infraction**				
No	35/508	1.47 (0.79-2.72)	0.224	0.085
Yes	18/287	3.00 (1.41-6.40)	0.004	

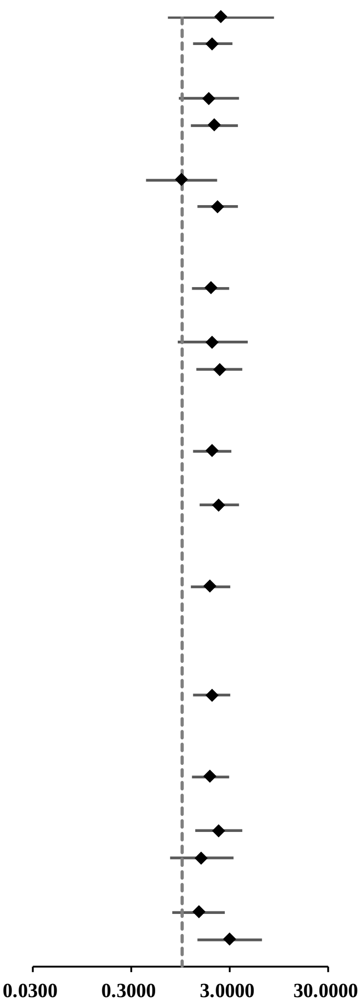

Fig. S2 Cox proportional hazards analysis between big ET-1 and secondary endpoints in various stratifications.
